# Simulating Microscale Urban Airflow and Pollutant Distributions Based on Computational Fluid Dynamics Model: A Review

**DOI:** 10.3390/toxics11110927

**Published:** 2023-11-13

**Authors:** Qian Liang, Yucong Miao, Gen Zhang, Shuhua Liu

**Affiliations:** 1 State Key Laboratory of Severe Weather & Key Laboratory of Atmospheric Chemistry of CMA, Chinese Academy of Meteorological Sciences, Beijing 100081, China; liangqian456@126.com (Q.L.); zhanggen@cma.gov.cn (G.Z.); 2 Department of Atmospheric and Oceanic Sciences, School of Physics, Peking University, Beijing 100871, China

**Keywords:** urban meteorology, microclimate, street canyon, air pollution, pollutant dispersion

## Abstract

Urban surfaces exert profound influences on local wind patterns, turbulence dynamics, and the dispersion of air pollutants, underscoring the critical need for a thorough understanding of these processes in the realms of urban planning, design, construction, and air quality management. The advent of advanced computational capabilities has propelled the computational fluid dynamics model (CFD) into becoming a mature and widely adopted tool to investigate microscale meteorological phenomena in urban settings. This review provides a comprehensive overview of the current state of CFD-based microscale meteorological simulations, offering insights into their applications, influential factors, and challenges. Significant variables such as the aspect ratio of street canyons, building geometries, ambient wind directions, atmospheric boundary layer stabilities, and street tree configurations play crucial roles in influencing microscale physical processes and the dispersion of air pollutants. The integration of CFD with mesoscale meteorological models and cutting-edge machine learning techniques empowers high-resolution, precise simulations of urban meteorology, establishing a robust scientific basis for sustainable urban development, the mitigation of air pollution, and emergency response planning for hazardous substances. Nonetheless, the broader application of CFD in this domain introduces challenges in grid optimization, enhancing integration with mesoscale models, addressing data limitations, and simulating diverse weather conditions.

## 1. Introduction

The United Nations Department of Economic and Social Affairs projects that by 2050, 68% of the global population will reside in urban areas [1], and this is expected to intensify various climate-related challenges, including heightened energy consumption, increased greenhouse gas emissions, intensified urban heat island effects, elevated air pollution, and greater susceptibility to extreme weather events. The Sixth Assessment Report from the IPCC underscores that cities not only serve as focal points for climate change impacts and vulnerabilities but also as pivotal arenas for climate change response [2]. Urban landscapes are marked by towering architectural structures, narrow thoroughfares, heightened population densities, and intensified anthropogenic emissions, all while being susceptible to ecological degradation and resource limitations. Consequently, the endeavor to construct sustainable cities emerges as a paramount priority in realizing the United Nations Sustainable Development Goals [3].

When contrasting urban environments with natural landscapes, it becomes evident that urban surface alterations wield substantial influences over localized energy exchange processes, giving rise to unique climatic and environmental conditions [4]. Within urban areas, particularly within the atmospheric boundary layer beneath the rooftops, known as the urban canopy layer, dynamic thermal processes are significantly shaped by the presence of buildings, resulting in microscale processes [5]. Urban microscale climate and meteorology typically operate on spatial scales measuring less than 1 km and with time scales often shorter than 1 day, which are notably smaller than the spatial and temporal scales of large and mesoscale atmospheric motions [6]. At the microscale, within a city, the interplay of the built environment, local climate, and human activities is complex, and its impacts are wide-ranging, including buildings’ energy consumption, the dispersion of air pollutants, environmental thermal comfort, and human health [7]. Therefore, conducting comprehensive research into the physical processes and evolving dynamics of an urban microscale climate and meteorology is of the utmost significance, which will not only contribute to the informed and scientific development of cities, but will also play a critical role in achieving environmental management goals and mitigating the adverse impacts of urbanization on climate change.

The principal methods of studying the urban microscale processes include conducting field observations and laboratory-based physical experiments (e.g., wind tunnel and water tank experiments), and the use of computational fluid dynamics (CFD) models. However, when it comes to conducting direct field observations within urban areas, challenges arise because of limited resources (e.g., specialized equipment and personnel) and site-specific characteristics [8]. Nevertheless, well-designed, scaled-down field experiments offer viable alternatives by allowing for the deployment of a greater number of sensors [9,10]. These experiments facilitate the examination of phenomena like the urban heat island effect, urban canopy ventilation, and the urban thermal environment [9,10,11,12]. In addition to field observations, laboratory-based physical experiments offer controlled environments, allowing for the isolation and manipulation of specific variables for in-depth analyses [13,14,15,16], but they may not fully capture the complexity and diversity of actual urban environments. In contrast, CFD provides distinct advantages in tackling the intricacies of urban environments, including three-dimensional (3D) wind patterns, turbulence, and matter dynamics [17]. This capability effectively complements the inherent limitations associated with field observations and laboratory experiments, and it enables a detailed examination of factors such as the building heat capacity and the street canyon aspect ratio, shedding light on their impacts on near-surface turbulence and temperatures within actual urban environments. By combining these three methods, a multifaceted approach to the study of the urban microscale climate and meteorology becomes possible.

CFD, as exemplified using software packages such as Ansys Fluent and OpenFoam (Open Field Operation and Manipulation), involves the solution of the Navier–Stokes system of equations through the use of discretization techniques. Among the discretization methods that are commonly employed, the finite volume method stands out for its prowess in maintaining conservation principles and preserving lucid physical interpretations, making it the most widely employed mesh discretization approach in CFD simulations [18]. Dealing with turbulence (Table 1) within the urban canopy presents a significant challenge in CFD simulations. While a direct numerical simulation (DNS) effectively handles turbulence problems at low Reynolds numbers [19], more commonly used indirect turbulence numerical simulation methods include large eddy simulation (LES), Reynolds-averaged Navier–Stokes equations (RANS), and detached eddy simulation (DES). The LES directly simulates large-scale eddies, with small-scale eddies represented through parameterization schemes, improving the computational efficiency while describing essential turbulence behaviors. LES is particularly suitable for predicting flow characteristics within single or densely packed building complexes on small scales, albeit it presents challenges when applied to the study of whole city [20]. RANS, characterized by a lower grid resolution, provides a time-averaged treatment of the Navier–Stokes equations, solving for individual mean motion variables. The different mathematical models for the Reynolds stress term give rise to various turbulence closure models [21], including the Spalart–Allmaras model in a one-equation mode, the standard k-ε model in a two-equation mode, the realizable k-ε model, and the SST k-ε model [22]. These two-equation turbulence models, which demand a minimal grid accuracy and offer swift convergence, are widely adopted to study wind field characteristics in urban microscale processes. The DES combines elements of both approaches, utilizing the RANS to predict the boundary layer turbulence and using the LES to simulate small-scale turbulent structures [23]. When selecting a turbulence scheme, considerations should encompass the physical phenomena, accuracy requirements, and available computational resource.

**Figure 1:**
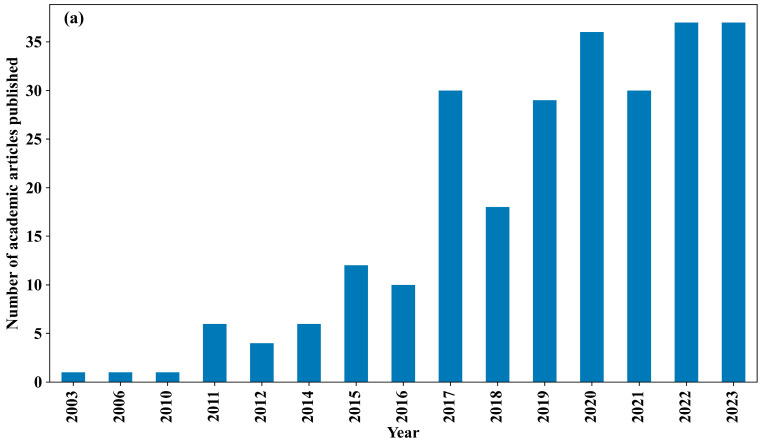

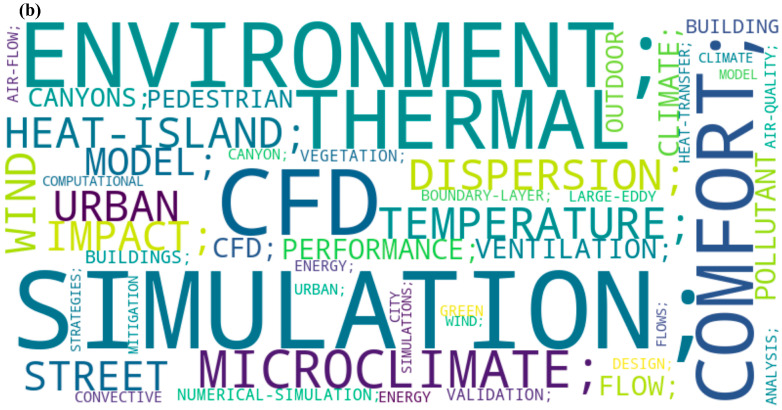
(**a**) Annual academic articles related to keywords ‘CFD, microclimate, and urban’ in the Web of Science database dating from 2003; (**b**) the keyword cloud diagram.

Overall, CFD offers flexible grid discretization, a range of turbulence modeling options, and user-friendly visualization tools, facilitating the comprehensive analysis of physical processes within intricate urban landscapes [24], and it serves as a powerful tool to translate theoretical principles into practical insights. Through simulations, it is possible to explore the specific impacts of parameters like the building heat capacity and street canyon aspect ratio on urban microscale processes, offering valuable knowledge for urban planning and environmental management. We conducted a search in the Web of Science database using the keywords ‘CFD, microclimate, and urban’ and identified 258 academic publications dating from 2003 (Figure 1a). Notably, approximately 84% of these publications emerged after 2016. When analyzing the frequency of these keywords across all of the papers (Figure 1b), it is evident that the primary focus of these publications lies in the CFD simulation process, urban environmental characteristics, and their effects on human comfort, particularly in the context of urban streets. While there are some review papers available that examine urban microclimate [25,26], these mostly offer evaluations of numerical methods without delving into CFD technology’s specificities or the thermal and dynamic physics of intricate urban surfaces. To address this gap, our review will primarily concentrate on numerical studies of microscale urban airflow and pollutant distribution utilizing CFD. The subsequent sections will provide an exhaustive summary of the research findings from urban microscale CFD simulations, encompassing both idealized scenarios and real-world conditions.

**Table 1:** Summaries of different CFD turbulence models.

Turbulence Models	Advantages	Disadvantages	Ref.
DNS	The DNS is far more accurate than any numerical method to solve the Navier–Stokes equations, and it is a useful tool in fundamental research on turbulence.	The computational cost of the DNS is very high, even at low Reynolds numbers.	Moin and Mahesh [19]
RANS	The RANS methods offer the most economic approach to compute complex turbulence, and they are suitable for many urban meteorology applications and typically provide the level of accuracy required.	The modeling assumptions used to derive the mathematical formulation limit the simulation accuracy.	Hussain et al. [22]
			Van Hooff and Blocken [27]
			Gao et al. [28]
			Blocken et al. [29]
			Baik and Kim [30]
			Flaherty [31]
LES	The LES is capable of handling flow instabilities and intermittencies and provides detailed information about turbulence structures.	The computational cost of LES is high. The LES models are primarily viewed as research tools rather than practical solutions for real urban meteorology applications.	Xie and Castro [32]
			Lim et al. [33]
			Buccolieri et al. [34]
DES	These methods are hybrid RANS-LES models, and they overcome some of the limitations of the RANS models and reduce computational cost compared to a fully fledged LES approach.	The DES may have inaccurate velocity and stress values at the RANS and LES interface.	Breuer et al. [23]

## 2. Idealized Simulations of Microscale Meteorological Processes

The urban landscape is a dynamic interplay of elements, featuring elongated street canyons, strategically positioned street trees, and buildings of varying heights [35]. To grasp the fundamental principles driving urban microscale processes, a common approach involves simplifying building configurations. This allows for the elucidation of fundamental laws through idealized CFD simulations, which may encompass scenarios with single buildings and 2D or 3D street canyons, as well as regular arrays of buildings.

**Figure 2:**
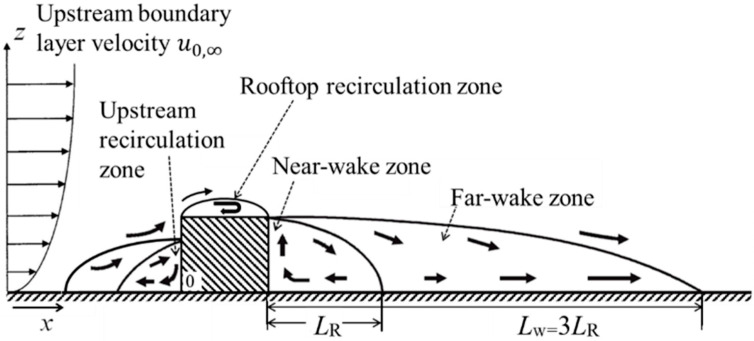
Schematic diagram of the flow pattern around an isolated building (*L*_R_ is the length of the near-wake zone, and *L*_W_ is the length of the far-wake zone) [36].

In the vicinity of a single building, the flow field (Figure 2) is characterized by distinct regions, including the upstream recirculation, rooftop recirculation zone, near-wake zone, and far-wake zone [36]. When multiple buildings encircle an individual structure, the flow fields intersect and interact, giving rise to intricate flow patterns both among the buildings and within the building array.

Street canyons, defined as narrow spaces enclosed by urban roads and adjacent buildings, represent an elemental unit of urban infrastructure. Street canyons serve as critical interfaces between indoor and outdoor environments, and they function as vital activity spaces for urban residents [37]. Assuming a street canyon to be infinitely long, its shape can be further simplified to a 2D state, and the flow field within is characterized by the presence and intensity of vortices, which are influenced by the geometric configuration of the canyon. Taller buildings within a street canyon possess the capacity to accelerate and guide winds, while shorter structures may cause the disruption and redirection of the flow. Additionally, increasing the aspect ratio can induce channeling effects, heightening the wind speeds and turbulence within the canyon, and leading to various flow field states, including isolated rough flow, wake interference flow, and climbing flow, within the street canyon [38]. With a fixed aspect ratio, higher background wind speeds amplify the vortex activity in the street canyon [30]. Furthermore, the roof’s structure also plays a crucial role in shaping the vortex patterns within the street canyon [39,40]. Various roof shapes, such as downwind wedges, upwind wedges, trapezoids, and circles, among others, as well as roof slopes, can impact the vortex morphology and the pollutant dispersion conditions in the street canyon [41,42,43,44].

Compared to 2D simulations, the utilization of 3D CFD simulations offers the capability of assessing more intricate meteorological conditions and building configurations, including variables like the ambient wind direction and building length. The simulation results for 3D street canyons indicate that deeper street canyons can lead to longer residence times for pollutants, allowing for enhanced secondary processes such as photochemical reactions [45], and asymmetrical street canyon layouts generally facilitate superior ventilation conditions compared to symmetrical configurations [46]. When buildings downstream of a street canyon are elevated, the airflow within the street canyon is obstructed, leading to an augmented downdraft on the windward side of these buildings [46], consequently intensifying the vertical vortex within the street canyon. Furthermore, the prevailing wind direction can significantly influence the wind characteristics within the 3D street canyon [47]. When the wind direction is perpendicular to the street canyon, it results in low wind speeds and poor ventilation, whereas an alignment of the wind direction parallel to the street canyon leads to elevated wind speeds and a more uniform pressure distribution [48,49]. In addition, the stability of the atmospheric boundary layer, which is used as an inflow condition in CFD simulations, also exerts an influence on the flow field within the 3D street canyon. Increased instability in the atmospheric boundary layer leads to amplified wind and turbulence fields within the street canyon [50]. Higher ground temperatures may lead to the formation of multiple vortex structures within the built-up areas, facilitating pollutant dilution and removal [51,52].

The recent upsurge in CFD studies focusing on urban greening expands our understanding of microscale meteorology in street canyons, significantly impacting urban areas. Street trees have notable impacts on the flow patterns within canyons [53], as they introduce an added surface roughness and act as physical wind obstructions, causing the airflow to bifurcate and circumvent the tree canopy, and they give rise to vortices and eddies within the flow. When positioned along the street canyon’s sides, tree canopies act as buffers, effectively reducing the wind speeds [54]. Optimizing the spacing between trees and the distances between trees and adjacent buildings may further enhance the ventilation within the street canyon [55]. The size of the tree canopy and the gaps between trees critically influence shading and, consequently, the thermal environment within the canyon [56]. Broad canyons benefit from street trees that are particularly adept at reducing wind speeds, while narrower ones enjoy the cooling and shading effects of these trees [57,58]. In practical scenarios, Buccolieri employed CFD to model wind fields and pollutant dispersion in vegetated street canyons, finding that the aerodynamic effect of vegetation on pollutant concentration varied with the wind direction, decreasing with higher aspect ratio in perpendicular wind conditions but notably increasing with inclined winds [34].

## 3. Realistic Simulations of Microscale Meteorological Processes

**Figure 3:**
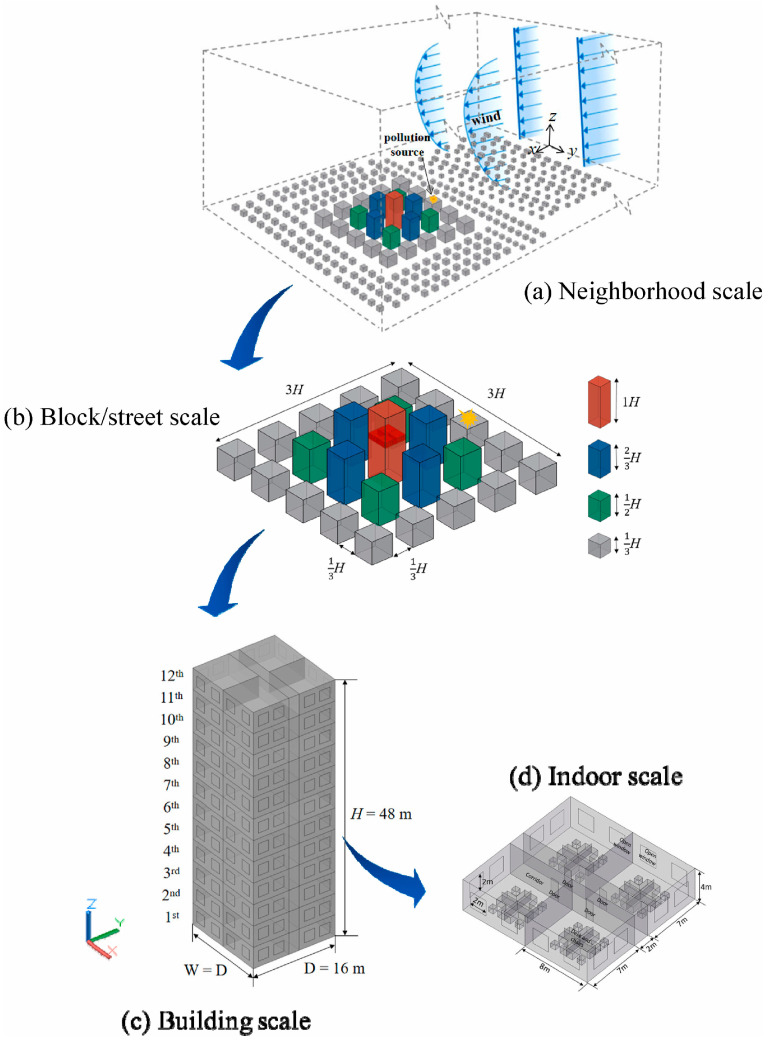
A multi-scale diffusion model of air pollutants passing through a building cluster from the outdoor environment into the indoor scale [62], including (**a**) neighborhood scale, (**b**) block/street scale, (**c**) building scale, and (**d**) indoor scale. D, W, and H are the length, width, and height of the target building, respectively.

Simulating urban microscale meteorological processes in realistic urban environments (Figure 3) is a complex endeavor that involves several interacting factors and presents a range of challenges, including geometrical/thermal heterogeneity [59,60], mesoscale interactions [61,62], and complex surface effects [63].

**Figure 4:**
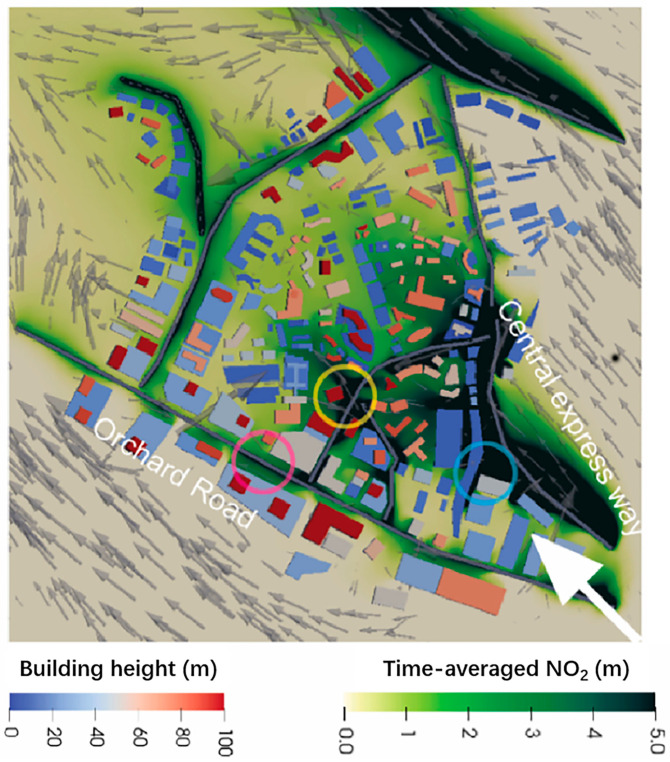
Simulated fields of NO_2_ concentration and wind vectors at the pedestrian level [65]. The blue-to-red shapes indicate the building heights, and the yellow-to-green shapes indicate the pollutant concentrations. The circles mark the pollutant accumulation points.

To engage in real simulations of urban microscale processes using CFD, it is imperative to establish appropriate initial and boundary conditions [31]. The ambient wind field and turbulence intensity within a simulation domain can be determined using a combination of actual observational data or fitted mathematical functions [64,65]. Flaherty and team designed the wind speed profile at the inlet boundary based on a fitted logarithmic law wind profile derived from empirical observations, and they found that low buildings exerted minimal influences, while a few high-rise structures significantly affected the transport and diffusion processes [31]. In downtown Singapore, high-resolution CFD simulations demonstrated that the heterogeneous urban morphology (i.e., the local building typology and height) significantly impacted the local pollutant concentration (Figure 4) [65]. Moreover, refining the computational grid, setting rational boundary conditions, and selecting appropriate turbulence models has enabled CFD to address various topics, including near-surface wind comfort [66], the design of the natural ventilation systems in urban areas [67,68,69], the analysis of indoor and outdoor air exchange dynamics [70,71], and the evaluation of wind power resources within urban settings [72,73].

These abovementioned CFD simulations relied on limited field data or theoretical wind profiles, affecting the precision and representativeness. The integration of CFD models with larger-scale meteorological models has significantly improved the provision of realistic starting parameters for CFD applications, enabling a more comprehensive understanding of the interactions between micro- and mesoscales [74]. For instance, in regions like Beijing [75], Hebei Province [76], and Shenyang [77], researchers have successfully simulated and studied wind and turbulence fields by integrating models at various scales. Tewari et al. demonstrated that coupling the Weather Research and Forecasting Model (WRF) with CFD can faithfully replicate wind field variations in the lower portion of the urban boundary layer [78], and they further highlighted that fine-tuning the parameterization scheme of the urban canopy within the WRF can further enhance the performance of the coupled model [78]. The multi-scale simulations reveal that an increased wind speed enhances the natural ventilation and air pollutant dispersion within built-up areas [79], while stable boundary layer conditions lead to pollutant retention around individual buildings [80]. Based on the simulations, it was recommended to implement strategies such as widening streets and adjusting the aspect ratio of street canyons to enhance local ventilation and facilitate more effective pollutant dispersion [81,82]. In the event of a sudden release of hazardous gases, there is a rapid and intense surge in the concentration, causing immediate and significant local variations within the urban landscape, driven by microscale meteorological processes [83]; coupled models have proven to be invaluable tools to gain comprehensive insights into flow fields, offering a cost-effective and secure approach for risk assessment [84].

**Figure 5:**
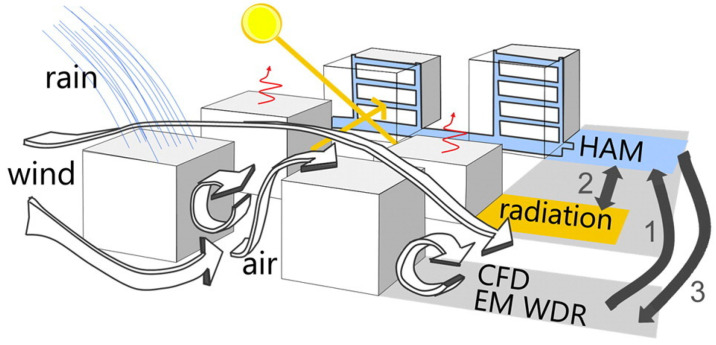
Schematic of the main physics implemented in the coupled urban microscale meteorological model. The air domain, modeling wind flow, and wind-driven rain (WDR) exchange information with the HAM (heat and moisture transport in porous media) model, which is iterated with the radiation model [91].

Moreover, within urban environments, the interplay of elements such as buildings, vegetation, and water bodies significantly shapes wind patterns and turbulence dynamics [85,86,87,88], while also influencing heat transfer dynamics [89]. This gives rise to dynamic, non-uniform heating patterns throughout the city, resulting in the delineation of distinctive urban microclimate characteristics, including the formation of urban heat islands, which, in turn, exert profound effects on the broader environmental context [90]. Understanding these intricate dynamics necessitates delving deeply into a multitude of complex physical processes, presenting an imposing challenge. Kubilay et al. employed integrated models (Figure 5), including the wind-guided precipitation model (WDR), the heat–air–moisture model (BE-HAM), and the radiation model, to dissect the intricate choreography of these processes within the urban environment [91]. Because urban heat islands are significantly affected by certain materials’ limited water-absorbing capacities and their affinity for capturing copious amounts of solar radiation, various strategies have been proposed to mitigate heat waves [92], such as converting pavements into permeable materials and applying high-albedo coatings [93]. Furthermore, the exchange of indoor and outdoor air is pivotal in shaping human living conditions (Figure 3). Currently, two primary approaches are utilized to simulate this exchange using CFD: (1) coupled simulation, which models both indoor and outdoor environments within the same computational domain, and (2) the regional decomposition method, where indoor and outdoor domains are discretized separately, with the outdoor data serving as boundary conditions for indoor simulation [85]. CFD simulations demonstrate that indoor airflow can be significantly influenced by external meteorological conditions [94]. Horan and Finn observed that an increase in the outdoor wind speed leads to a linear rise in the indoor air change rate, while the impact of the outdoor wind direction hinges on the configuration of building vents [71].

In recent years, the integration of machine learning and artificial intelligence techniques with CFD has revolutionized studies in urban meteorology [95,96,97,98], which can help refine turbulence models and capture the effects of urban surface heterogeneity. For example, Ding et al. employed machine learning techniques to enhance the CFD model for indoor–outdoor coupling, introducing a comprehensive index for the swift assessment of ventilation in urban planning and design [95]. Mortezazadeh et al. combined machine learning and CFD to achieve precise predictions of wind speeds in urban areas, evaluating the potential for wind power utilization [96]. Additionally, Javanroodi et al. proposed a hybrid model that combines CFD with artificial neural networks in simplified urban settings, which was trained using multilayer perceptrons and deep neural networks, and they demonstrated improved accuracy in predicting urban microscale wind fields [99].

In summary, the exploration of the relationship between the urban microscale meteorological processes and the physical attributes of the underlying surface is of significant importance to enhance neighborhood air quality and improve the comfort of human settlements [100,101,102,103,104]. However, the majority of existing studies in this field primarily focus on meteorological conditions characterized by abundant sunshine and low humidity. Enhancing the parameterization of urban underlying surface properties in complex meteorological conditions and precisely depicting their interactions with the surrounding environment in CFD simulations require further refinement. 

## 4. Conclusions

CFD has achieved a high level of maturity and is extensively utilized to investigate urban micrometeorological processes, encompassing not only idealized city simulations but also the intricate flow and pollution patterns within real urban environments. Significant variables such as the aspect ratio of street canyons, building geometries, the ambient wind direction, atmospheric boundary layer stabilities, and street tree configurations play crucial roles in influencing microscale physical processes and the dispersion of atmospheric pollutants. The integration of CFD with mesoscale meteorological models and cutting-edge machine learning techniques enables the high-resolution, precise simulation of urban meteorology, laying a scientific foundation for sustainable city development and air pollution mitigation. Nonetheless, its further application is accompanied by several challenges. Firstly, while CFD offers flexibility, the diverse range of turbulence calculation methods lacks established best practices, posing difficulties in balancing computational efficiency, resource demands, and model performance. Secondly, issues like grid mismatches and variations in discretization methods can complicate data interpolation and information transfer, potentially affecting the accuracy and stability of simulation results when integrating CFD with mesoscale models. Thirdly, the limited spatial distribution and the quantity of data points from field observations and wind tunnel experiments pose challenges in validating the CFD simulation results. Lastly, it is imperative to conduct further research to advance CFD capabilities of simulating a wider array of weather conditions, encompassing scenarios like heavy rain, heatwaves, hurricanes, and other extreme events.

## Data Availability

The data presented in Figure 1 is sourced from Web of Science (https://www.webofscience.com/wos/alldb/basic-search, accessed on 31 October 2023).
